# Potential accessibility scores for hospital care in a province of Japan: GIS-based ecological study of the two-step floating catchment area method and the number of neighborhood hospitals

**DOI:** 10.1186/s12913-017-2367-0

**Published:** 2017-06-26

**Authors:** Takashi Nakamura, Akihisa Nakamura, Kengo Mukuda, Masanori Harada, Kazuhiko Kotani

**Affiliations:** 1 Center for Community Medicine, Jichi Medical University, 3311-1 Yakushiji Shimotsuke, Tochigi, 3290498 Japan; 2 Gero Municipal Osaka Clinic, 1965 Ohshima Osaka Gero, Gifu, 5093106 Japan; 3 Internal Medicine, Nichinan Hospital, 511-7 Shoyama Nichinan Hino, Tottori, 6895211 Japan; 4 Department for Support of Rural Medicine, Yamaguchi Grand Medical Center, 77 Ohsaki Hofu, Yamaguchi, 7478511 Japan

**Keywords:** Potential accessibility, Two-step floating catchment area method, Geographical information system, Distance decay, General practice, Medically underserved area

## Abstract

**Background:**

For achieving equity of the accessibility to primary healthcare, measuring potential geographical accessibility is essential. The provider-to-population ratio is the most frequently used measure. However, it is difficult to be used in closer region because it does not take into consideration the people and health services beyond its boundary. In order to overcome this problem, we measured the potential access to hospital, using both distance measures and the enhanced two-step floating catchment area (E2SFCA) method. The aim of this study was to compare the number of hospitals in the neighborhood and the E2SFCA score with regard to the amount and equity for access to hospitals.

**Methods:**

This descriptive study used publicly available data from 2010. The E2SFCA score and number of neighborhood hospitals were obtained from Tochigi province in Japan using a geographic information system. Dataset of four measures by each census tract was obtained. The measures were E2SFCA score, number of hospitals within the 5 km range, number of hospitals within the 10 km range, and number of hospitals within the 15 km range. Correlation and disparity analyses with the Lorenz curve and Gini coefficient were performed.

**Results:**

The measures were obtained in a smaller area than municipality considering adjacent areas using a geographical approach. The E2SFCA score was 5.3 [3.2–7.3] hospitals/million (median [quantile range]), compared to 5.6 hospitals/million in total for the given district. The median number of hospitals within the 5 km, 10 km, and 15 km ranges were 1, 39, and 47, respectively. There was no hospital within the 5 km range in one third of the blocks. Both the number of hospitals within the 10 km range and those within the 15 km range were well correlated. Regional difference became smaller as the distance to count the number of hospitals increased. The gap between small number of hospitals and the high E2SFCA score indicated the location of community hospital in depopulated areas.

**Conclusions:**

The E2SFCA method is superior for analyzing spatial access to hospital, because it provides information in the closer sub-regions. Regional differences were hardly seen in access to hospital beyond the 10 km range. Further studies in other regions and countries are needed for precise assessment.

## Background

Maximizing and achieving equity of access to primary healthcare are prerequisites for achieving health. The vast majority of aged population was within close proximity to hospital facilities in Illinois, US [1]. However, inaccessibility of hospital services may increase the risk of asthma mortality [2] and overuse of cesarean delivery [3]. Accessibility to hospital care is one of the determinants of health.

“Access to healthcare” is often mentioned. However, there are complexities in the concept of access to primary healthcare [4]. The determination of “access to healthcare” remains unclear. Penchansky and Thomas mentioned five dimensions of access, including availability, accessibility, accommodation, affordability, and acceptability [5]. In this study, we focused on the spatial [6] or geographical profile of potential accessibility. Potential accessibility does not account for the realized utilization of medical care [7]. In the context of the medically underserved, whether healthcare is present at a site is important, as well as its utilization.

Measurements of the geographical profile of potential accessibility have some variations. Distance and travel time measures are two of the simplest ways of measuring potential access. Density measures including Kernel estimates of service density, two-step floating catchment area method are also known [8]. The two-step floating catchment area (2SFCA) method has been proposed as an example of a catchment model [9]. Thus, both distance measures and 2SFCA method have been employed in this study.

Recent literature concerned with the metrics of access to healthcare has focused on 2SFCA approaches, which are essentially a specialized form of the gravity model [10]. The 2SFCA method uses point features to represent the location of services and demand. In the first step, a distance catchment is placed around each primary healthcare service provider and a provider-to-population ratio is computed using the number of providers and estimated population falling within the area. In the second step, a similar floating catchment is placed over each demand center and the service accessibility for this population is rated by summing all provider-to-population ratios contained within the zone.

For the earliest version of the 2SFCA model, there are many criticisms, such as its reliance on a finite catchment size [11]. To address the concerns, an enhanced two-step floating catchment area (E2SFCA) method was proposed involving the introduction of a distance decay function into the floating catchments of both algorithmic steps [12]. The E2SFCA method is available for measuring spatial accessibility to primary care physicians [12, 13]. An increasing number of reports on geographical accessibility to primary healthcare are available from different countries, including China [14, 15], US [16, 17], Canada [18], Australia [19–21], India [22], Japan [23], and some other countries. Harada et al. showed the equity of accessibility using the Lorenz curve and Gini coefficient of the number of hospitals within certain ranges [24].

The aim of this study was to compare the E2SFCA score and the number of neighborhood hospitals with regard to both amount and equity.

## Methods

### Design

The present study is a descriptive study using a geographical information system with publicly available data from 2010.

### Subject area

The administrative census mesh block (“*Cho-cho-aza*”) in Tochigi province in Japan was set as the subject area. The population was 2,007,683, with 110 hospitals in 2010. The total number of hospital per 100,000 was 5.5.

### Data source

Population and the location of each block were extracted from ArcGIS data collection standard pack 2015 (Esri Japan Corporation, Tokyo, Japan). This database included data from Census 2010. The location of hospitals in 2010 was extracted from the National Land Numerical Information download service of the Land, Infrastructure and Transportation Ministry. The road network data in 2010 was extracted from the ArcGIS data collection road network, which included data from the Road Traffic Census 2010.

#### Distance from each block to each hospital

Every subject block was included, and every hospital in both Tochigi province and the neighboring provinces was included. Therefore, we accounted for the neighboring population beyond the border of Tochigi province. The distance from each block to each hospital was calculated using the Origin-Destination Cost Matrix command of ArcMap 10.4.1 (Esri, Redlands, CA, USA), and data, including the location of each block, location of each hospital, and road network information, were used. Thus, the distance data matrix of each block according to the hospital was obtained.

#### Distance measures: Number of neighborhood hospitals

According to the distance between a block and hospital, the number of hospitals within the 5 km range from each block was determined. The number of hospitals within the 10 km range and the number of hospitals within the 15 km range were similarly calculated. The majority of the aged are within a 7.7 km range of a hospital and an 18.6 km range of two hospitals in Illinois [1]. The distance decay function from the 5 km to 15 km distance was used to generate rural primary care access [20]. Therefore, 5 km, 10 km, and 15 km ranges were set for this measure.

#### E2SFCA method

According to the distance between a block and hospital, the weighing coefficient of distance decay was assessed (Eq. 1). The number of hospitals within the 5 km range was weighed as 1, the number of hospitals beyond the 15 km range was weighed as 0, and the number of hospitals between the 5 km and 15 km ranges was weighed as a coefficient with distance decay [20].

Equation 1 Weighing coefficient formula according to distance

1 $$ {W}_{i, j}=\left\{\begin{array}{c}1\kern1.75em ,\kern2.75em {d}_{i, j}<5\\ {}{\left(\frac{15-{d}_{i, j}}{15-5}\right)}^{1.5},5\le {d}_{i, j}\ \le 10\\ {}0\kern2.5em ,\kern0.5em 10\le {d}_{i, j}\end{array}\right. $$

where *d*_*i* , *j*_ represents the distance [km] between block *i* and hospital *j*.

In the first step, the catchment population of each hospital was calculated. The population in each block was weighed by distance decay, and the sum of the population according to each hospital was obtained (Eq. 2).

Equation 2 Step 1: Catchment population around each hospital

2 $$ {P}_j={\sum}_i{W}_{i, j}\times {P}_i $$

where *P*_*j*_ represents the neighborhood population around hospital *j*, *W*_*i* , *j*_ represents the weighing coefficient, and *P*_*i*_ represents the population within block *i*.

In the second step, the providers to the catchment population in each block were calculated. The reciprocal of the catchment population of each hospital was weighed by the distance decay, and the sum of its ratio according to each block was the E2SFCA score (Eq. 3).

Equation 3 Step 2: E2SFCA score

3 $$ {E2 SFCA}_i={\sum}_j\frac{W_{i, j}\times {S}_j}{P_j} $$

where *E*2*SFCA*_*i*_ represents the E2SFCA score of block *i*, *P*_*j*_ represents the neighborhood population around hospital *j*, *W*_*i* , *j*_ represents the weighing coefficient, and *S*_*j*_ represents the number of providers in hospital *j*. With regard to the number of hospitals, *S*_*j*_ = 1.

### Statistical analysis

The dataset of four measures according to each census tract was obtained with the procedure above. The measures were the number of hospitals within the 5 km range, number of hospitals within the 10 km range, number of hospitals within the 15 km range, and E2SFCA score. To investigate the relationship among the four measures, we performed correlation analysis using Spearman’s rank-order correlation. Then, to investigate disparity among the four measures, we performed disparity analysis using the Lorenz curve and the Gini coefficient. The Gini coefficient ranges from 0 to 1. The Gini coefficient 0 represents no disparity, and the Gini coefficient 1 represents maximum disparity. Stata/SE 14.2 (StataCorp, College Station, TX, US) was used for all statistical analyses. The alpha error was set to 0.05 for significance.

## Results

### Characteristics of the measures

There were 2583 census blocks, including 46 blocks with no residents. The median population of each block was 456, and the median area was 0.60 km^2^. The numbers of hospitals within the 5 km, 10 km, and 15 km ranges were 1, 39, and 47, respectively. The number of blocks where there was no hospital within the 5 km range was 825 (32.0%). There were at least 9 hospitals within the 10 km range. Total number of hospitals per 100,000 people (5.6) and the E2SFCA (5.3 [3.2–7.3], median [quantile range]) were almost the same (Table 1).

**Table 1:** Population, the number of neighborhood hospitals, and the E2SFCA score

	N	Median	(Quantile range)
Population	2583	456	(199–879)
Area (km^2^)	2583	0.60	(0.14–2.27)
Number of hospitals within the 5 km range	2583	1	(0–4)
Number of hospitals within the 10 km range	2583	39	(32–51)
Number of hospitals within the 15 km range	2583	47	(38–58)
E2SFCA score	2583	5.3	(3.2–7.3)

### Correlation among the measures

**Fig. 1:**
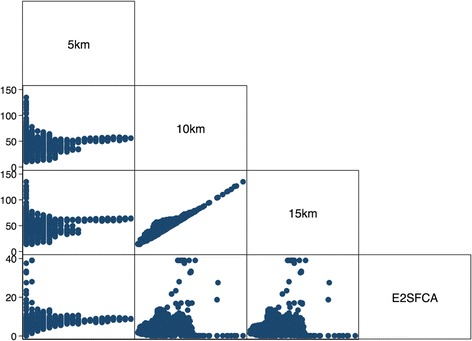
Scatter plot of the number of neighborhood hospitals and the E2SFCA score. Footnote: 5 km, number of hospitals within the 5 km range; 10 km, number of hospitals within the 10 km range; 15 km, number of hospitals within the 15 km range; E2SFCA, E2SFCA score

Correlation analyses among the different variables were performed. A significant correlation was noted between the E2SFCA score and the number of hospitals within the 5 km range (Spearman’s ρ = 0.696, *p* < 0.01) (Table 2), although a scatter plot showed limited tendency (Fig. 1). It suggests that each measurement reflects another aspect of the potential accessibility to hospital. Additionally, a significant positive correlation was noted between the number of hospitals within the 10 km range and the number of hospitals within the 15 km range (Spearman’s ρ = 0.898, *p* < 0.01) with linear correlation in the scatter plot (Fig. 1). It suggests that both the number of hospitals within the 10 km range and those within the 15 km range reflected similar aspect of the potential accessibility to hospital.

**Table 2:** Spearman’s correlation analysis

	Population	5 km	10 km	15 km
5 km	−0.266 (*p* < 0.01)			
10 km	−0.112 (*p* < 0.01)	0.142 (*p* < 0.01)		
15 km	−0.002 (n.s.)	0.225 (*p* < 0.01)	0.898 (*p* < 0.01)	
E2SFCA	0.066 (*p* < 0.01)	0.696 (*p* < 0.01)	0.344 (*p* < 0.01)	0.278 (*p* < 0.01)

Multi-comparisons were corrected with Bonferroni correction. Footnote: n.s., *p* > 0.05; 5 km, the number of hospitals within the 5 km range; 10 km, the number of hospitals within the 10 km range; 15 km, the number of hospitals within the 15 km range; E2SFCA, E2SFCA score.

### Disparity among the measures

**Fig. 2:**
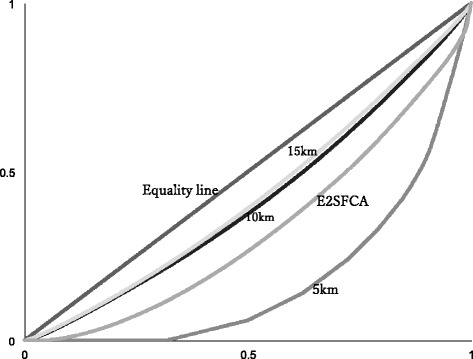
Lorenz curve of the regional distribution of the accessibility scores. The percentage of regional blocks is plotted on the x-axis, and the percentage of the accessibility scores in plotted on the y-axis. The diagonal line is the line of equality. A greater distance from the line of equality indicates a higher disparity in the regional distribution of the accessibility scores. Footnote: 5 km, number of hospitals within the 5 km range; 10 km, number of hospitals within the 10 km range; 15 km, number of hospitals within the 15 km range; E2SFCA, E2SFCA score

The Gini coefficient of each variable was calculated. The Gini coefficient became smaller as the distance for counting the number of hospitals increased. The Gini coefficient of E2SFCA score and Lorenz curve were within the range between the coefficient of the number of hospitals within the 5 km range and that of the number of hospitals within the 10 km range (Table 3, Fig. 2).

**Table 3:** The Gini coefficients from the Lorenz curve

	The Gini Coef
Number of hospitals within the 5 km range	0.645
Number of hospitals within the 10 km range	0.175
Number of hospitals within the 15 km range	0.150
E2SFCA score	0.348

### Geographical distribution

**Fig. 3:**
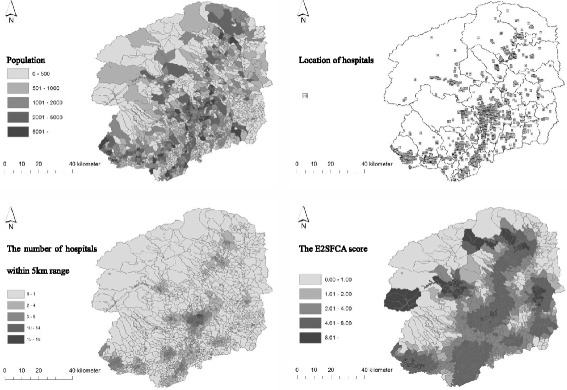
Geographical distribution. Distribution of the population (*left upper*), hospitals (*right upper*), number of hospitals within the 5 km range (*left lower*), and E2SFCA score (*right lower*). In the central part of the given area, both population and hospitals were aggregated. Despite the small number of hospitals, the high E2SFCA score indicates the presence of community hospitals in depopulated areas

The geographical distribution maps of the population, locations of hospitals, number of hospitals within 5 km, and E2SFCA score are presented in Fig. 3. In the central and the south region, there were many hospitals and the E2SFCA score was high. In the north region, the E2SFCA score was high, though the number of hospitals was small. This was because there were community hospitals in depopulated regions.

## Discussion

In this study, we compared access to hospital in a province of Japan with two measures, i.e. E2SFCA score and the number of hospitals within certain distance ranges. With conventional measures such as the number of hospitals per the number of people in the region, it was difficult to use in smaller area units than municipalities. The number of hospitals per population in the target district approximated the average of E2SFCA scores calculated for each sub-region (5.3 hospitals/million persons). There were different characteristics between E2SFCA score and the number of hospitals within certain distances. There were no hospitals within the 5 km range in one third of the blocks. There was a strong correlation between the number of hospitals within the 10 km range and the number of hospitals within the 15 km range.

The number of hospitals within certain distance ranges may reflect healthcare opportunities for each resident. The number of hospitals increases as the distance range increases, but there was little difference between the number of hospitals within the 10 km range and the number of hospitals within the 15 km range. Although the Japanese law limits the number of beds for each medical district consisting of one or more municipalities, the location of the hospital follows an economic principle. Aoki et al. showed that the appropriate area was within the 15–20 km range from the hospital in an ecological study of the number of patients seeking hospitals [25]. The regional distance of seeking hospitals among Japanese people might be around 10 km.

E2SFCA score reflects the balance between the number of hospitals and the population. One of the simplest measures for this balance is the number of hospitals per the number of people in the region, within a certain range. However, this conventional measure does not take into consideration the population or hospitals outside the boundary; it does not work well in the setting of narrower regional units. E2SFCA method made it possible to evaluate access to healthcare in narrower area unit by considering neighborhood areas. Furthermore, since the score approximates to the conventional indicator, it is easy to understand intuitively. It would be used to evaluate the localization of the poorer accessibility to hospital care.

The present study has some limitations. Spatial potential accessibility to hospital care was assessed in the current study. The catchment area in the real world differs from hospital to hospital [21, 26]. Additionally, the traffic situation may affect accessibility [27]. A number of variation of the 2SFCA method have been devised, including the optimized 2SFCA method with accounting for the number of realized visiting patients [18], commuter-based version of the 2SFCA method [28], three-step floating catchment area method [29], modified 2SFCA method [30, 31], enhanced variable two-step floating catchment area (EV2SFCA) method [32], and multi-criterion two-step floating catchment area method [33]. Another formula of the distance decay used in this study has been proposed [11]. Furthermore, this study was performed in a certain district of Japan. As mention above, the accessibility to healthcare could be affected by county-specific social background factors, including traffic conditions and the healthcare system. Further studies in other regions and other countries are needed for precise assessment.

## Conclusions

The E2SFCA method is superior for analyzing spatial access to hospital, because it would provide information in the closer sub-regions with the approximate value and the same unit dimension as conventional provider-to-population ratio measure. Regional differences were hardly seen in access to hospital beyond the 10 km range.

## Abbreviations

2SFCA: Two-step floating catchment area
E2SFCA: Enhanced two-step floating catchment area
